# Selenium-Containing Agents Acting on Cancer—A New Hope?

**DOI:** 10.3390/pharmaceutics15010104

**Published:** 2022-12-28

**Authors:** Sabrina Garbo, Silvia Di Giacomo, Dorota Łażewska, Ewelina Honkisz-Orzechowska, Antonella Di Sotto, Rossella Fioravanti, Clemens Zwergel, Cecilia Battistelli

**Affiliations:** 1 Department of Molecular Medicine, Sapienza University of Rome, Viale Regina Elena 324, 00161 Rome, Italy; 2 Department of Physiology and Pharmacology “V. Erspamer”, Sapienza University of Rome, Piazzale Aldo Moro 5, 00185 Rome, Italy; 3 Department of Technology and Biotechnology of Drugs, Faculty of Pharmacy, Jagiellonian University Medical College in Kraków, Medyczna 9, 30-688 Kraków, Poland; 4 Department of Drug Chemistry and Technologies, Sapienza University of Rome, Piazzale Aldo Moro 5, 00185 Rome, Italy

**Keywords:** selenium, cancer, cancer therapy, immunotherapy, small organic Se-containing compounds, Se-nanoparticles

## Abstract

Selenium-containing agents are more and more considered as an innovative potential treatment option for cancer. Light is shed not only on the considerable advancements made in understanding the complex biology and chemistry related to selenium-containing small molecules but also on Se-nanoparticles. Numerous Se-containing agents have been widely investigated in recent years in cancer therapy in relation to tumour development and dissemination, drug delivery, multidrug resistance (MDR) and immune system-related (anti)cancer effects. Despite numerous efforts, Se-agents apart from selenocysteine and selenomethionine have not yet reached clinical trials for cancer therapy. The purpose of this review is to provide a concise critical overview of the current state of the art in the development of highly potent target-specific Se-containing agents.

## 1. Introduction

In the early 19th century, Jöns Jacob Berzelius discovered selenium (Se), a chalcogen that belongs to group 16 along with oxygen, sulphur, tellurium, and polonium [1]. It is widely believed that elementary Se and compounds containing Se can cause mental illness, cancer, and other diseases [2]. In the 1970s, D. Forst discussed the, at that time, confusing effects of Selenium in cancer biology in his critical paper, coining the term “Selenophobia” [3]. However, today, it is well accepted that Se has both protective and toxic effects on organisms, such as the nervous system and the heart [2,4]. Se can safely be incorporated into a variety of organic compounds for the treatment of various diseases [2,4].

Chemistry-wise, Se has several oxidation states and can be oxidized and reduced by redox agents more easily than sulphur (S). Thus, Se can act as a nucleophilic or electrophilic agent by donating or accepting electrons [1]. Since Se behaves in this way, it is suitable for use in chemistry and biology. Medicinal chemistry, biochemistry, and biology are all interconnected areas where selenium and, specifically, seleno-compounds are considered promising candidates for drug discovery [4]. Since the 1980s, a variety of selenium-containing compounds have been described to exhibit chemopreventive and antioxidant properties with a number of positive outcomes [5]. As a result, considerable advancements have been made in understanding this element’s complex biology and chemistry, showing that Se plays a crucial role in various biological processes [2,6]. It can also exert toxic effects [2,6].

Selenoproteins (SePs) are particularly interesting, as they impact the health of many organs by maintaining broader homeostasis through regulation of the thyroid hormone level, endoplasmic reticulum stress and antioxidant defence [7], but also immune and inflammatory responses. The unique nature of selenium is that it is folded into its protein while the protein molecule is still being made [8]. SePs carry the rare selenium-containing selenocysteine **1** residue. This is of great importance, as it means that the incorporation of this 21st amino acid, selenocysteine **1**, is genetically regulated, and through this process, it is possible to modify the activity of different SePs. Among the roughly 40 Se-dependent proteins discovered thus far with a variety of biological functions, glutathione peroxidase (GPx) [9,10] is the most studied and explored. 

The purpose of this review is to provide a concise overview of selenium-containing small molecules as well as Se-nanoparticles, which have been widely investigated in recent years in cancer therapy, spanning from tumour development and dissemination, drug delivery to multidrug resistance (MDR) and immune system-related (anti)cancer effects.

## 2. Therapeutic Properties of Selenium as a Trace Element 

**Figure 1:**
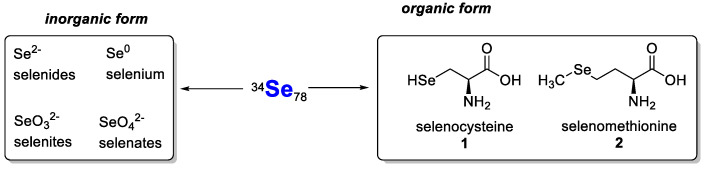
The most important inorganic and organic forms of selenium [11].

**Figure 2:**
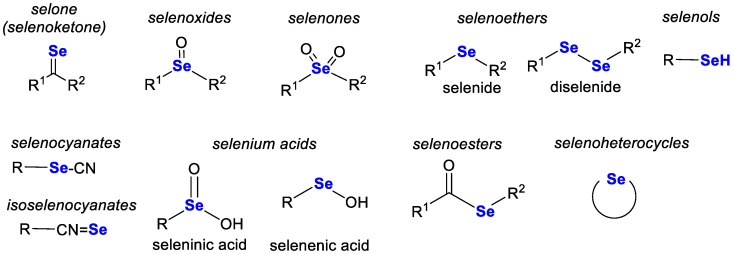
General formulas of major organoselenium compounds [11,13].

Selenium is a solid non-metal with acidic properties (chemically resembling sulphur) that occurs essentially in numerous allotropic varieties. It occurs in nature and in organisms in two forms (inorganic and organic) and in four oxidation states: −II, 0, +IV and +VI. The most important inorganic and organic forms of selenium are shown in Figure 1 [11]. Inorganic selenium can be found in minerals in the forms of selenides (most commonly), selenates and selenites [12]. Selenium minerals are extremely rare. Only 123 such species have been described thus far [12]. In nature, organically bound selenium mainly occurs in selenoamino acids (mainly), selenopeptides and SePs [11]. Other organoselenium compounds may belong to various chemical groups. The most important forms are shown in Figure 2 [11,13]. Such compounds are usually characterised by higher bioavailability and lower toxicity than inorganic compounds.

The incorporation of selenium into various organoselenium compounds (synthetic and natural) often results in beneficial pharmacological activity. The type of selenium linkage in an organic molecule significantly influences the biological activity of these compounds, i.e., whether they exhibit a beneficial effect (reactive oxygen species (ROS) uptake, oxidative capacity) or a toxic effect (ROS induction or release of elemental Se). The biological activity of selenium compounds is mainly related to antioxidant properties, i.e., the ability to scavenge ROS, and depends on the ability of compounds to convert into the corresponding selenols and on the rate at which this process takes place. Selenols show the most potent antioxidant properties among selenium derivatives. They readily dissociate to form the RSe^−^ anion at physiological pH and are active in a GPx-like catalytic cycle [13]. The cytotoxic effect of organic selenium compounds relates to their ability to release elemental Se (0) that can interact with lipoproteins to form cytotoxic cysteine●selenium conjugates. This effect is usually missing in selenium heterocycles with selenium in sp^2^ hybridisation, as such compounds are stable and do not show the ability to release Se (0) [13].

Selenium is an essential micronutrient needed for the proper functioning of the body. It is a component of two key amino acids: selenocysteine **1** and selenomethionine **2**, which, in turn, build up essential enzymes for the human body: glutathione peroxidases (GPxs), thioredoxin reductases, iodothyronine deionidases or SePs such as selenoprotein O, selenoprotein P or selenoprotein R [14,15]. SePs are involved in many metabolic and functional pathways, and most of them catalyse oxidation–reduction reactions. SePs P, mainly present in plasma, are also highly expressed in the brain. They are thought to be transporters of selenium from the liver to the brain [16], and recent studies have shown their involvement in the pathogenesis of diseases such as Alzheimer’s and Parkinson’s [17,18].

Selenium can be classified as a hermetic element because it is beneficial at low doses and harmful at high doses. Both of these conditions (deficiency and excess) are bad and can eventually lead to health problems, causing certain diseases and even death, especially when they occur over a long period. The main health problems that can arise from selenium deficiency or excess are shown in Table 1 [19,20,21]. In some cases, both selenium deficiency and excess can lead to the same health problems, i.e., increased mortality, type 2 diabetes or prostate cancer [19]. Selenium deficiency may affect more than one billion people worldwide and especially affects the expression of SePs in the body [22]. Thus, selenium deficiency can affect life expectancy, accelerating the ageing process or increasing susceptibility to immunological or cancerous diseases [21] and causing various cardiovascular diseases, including cardiomyopathies [7]. Moreover, lower (than in healthy) selenium levels were observed in patients with a more severe course of COVID-19 (SARS-CoV-2) [23]. Plasma selenium amount is thought to be the most reliable measurement of selenium levels in the body. It is considered that the optimum selenium concentration should be between 90 and 120 µg/L [24]. It is difficult to clearly determine what level of selenium is toxic, as the threshold of selenium toxicity may depend on genetic and environmental conditions and, above all, on the duration of the condition.

According to the WHO, the recommended daily intake of selenium should be between 55 and 70 µg/day for adults and 25 µg/day for children [25]. The amount also depends on (despite age and gender) the area of residence. In selenium-deficient areas (e.g., South-East China, the northern-east part of Europe [25,26]), much higher daily supplementation is recommended. The upper tolerable nutrient intake level recommended in many countries is 400 µg/day [27].

Selenium is mainly supplied to the body through food of plant origin but also from animal and fungal sources. The foods most rich in selenium are yeast, Brazil nuts, Shiitake mushrooms, garlic, onion, red meats, eggs, seafood or broccoli [21]. In the tissues of plants and animals, selenium occurs as organic selenium in the form of selenocysteine **1** and selenomethionine **2** [24]. The bioavailability of selenium present in food also depends on dietary factors such as fat, protein and heavy metal content.

**Figure 3:**
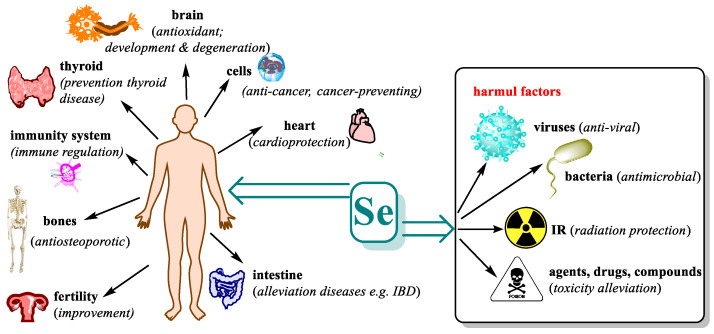
Effect of selenium on various organs and functions. (IBD, inflammatory bowel disease; based on references: [20,24,28,29,30,31,32,33,34,35,36,37,38]).

Research in recent years has shown that selenium has beneficial and protective effects in several disorders and disease states, but often the exact mechanism of these effects is not known and requires further investigations to clarify. The therapeutic utility of selenium has been studied in many directions, both in vitro and in vivo studies and even in clinical trials. Figure 3 shows the most important systems and organs that are beneficially affected by adequate levels of selenium and which adverse effects it can mitigate.

## 3. Selenium-Containing Compounds as Therapeutic Agents 

Selenium (Se)-containing compounds has attracted great attention in the past years due to their promising bioactivities, including antioxidant, antimicrobial, antiviral, antiparasitic, anti-inflammatory, neuroprotective, and anticancer ones [39,40]. Selenites, especially selenite **3** and its sodium salt **3a**, are known to undergo oxidation and reduction reactions, leading to the generation of their divalent cations (Se^2+^), endowed with oxidant properties [41]. Sodium selenite **3a** is also considered a broad-spectrum anti-bacterial agent and a promising adjuvant strategy to overcome antibiotic resistance [42]. Moreover, selenomethionine **1** has been considered as a potential candidate for the treatment of coronavirus infections owing to its antioxidant and immunomodulatory properties [43,44]. Lastly, ebselen **4** and its derivatives exhibited neuroprotective effects against different toxicants, thus suggesting a possible future interest in managing mental disorders and degenerative diseases [45,46,47].

In the years, special attention has been devoted to the potential usefulness of Se-containing compounds in the battle against cancer, acting as anticancer and chemopreventive agents, inducing antioxidant and pro-oxidant effects at low and high doses, respectively [48,49]. Inorganic compounds, especially selenite **3**, showed chemopreventive properties higher than the organic ones, despite their higher toxicity [50]. Conversely, diverse organic compounds belonging to the selenide, diselenide, cyanate, urea and ester groups [51,52] were low-toxic and endowed with pleiotropic anticancer properties [51,52]. Apoptosis represents the primary mechanism by which Se-containing compounds exploit their anticancer activity in cells. However, other non-apoptotic cell death mechanisms, including cell cycle arrest, necrosis, autophagy, ferroptosis, necroptosis, entosis, anoikis, NETosis, or mitotic catastrophe, have also been reported. 

**Figure 4:**
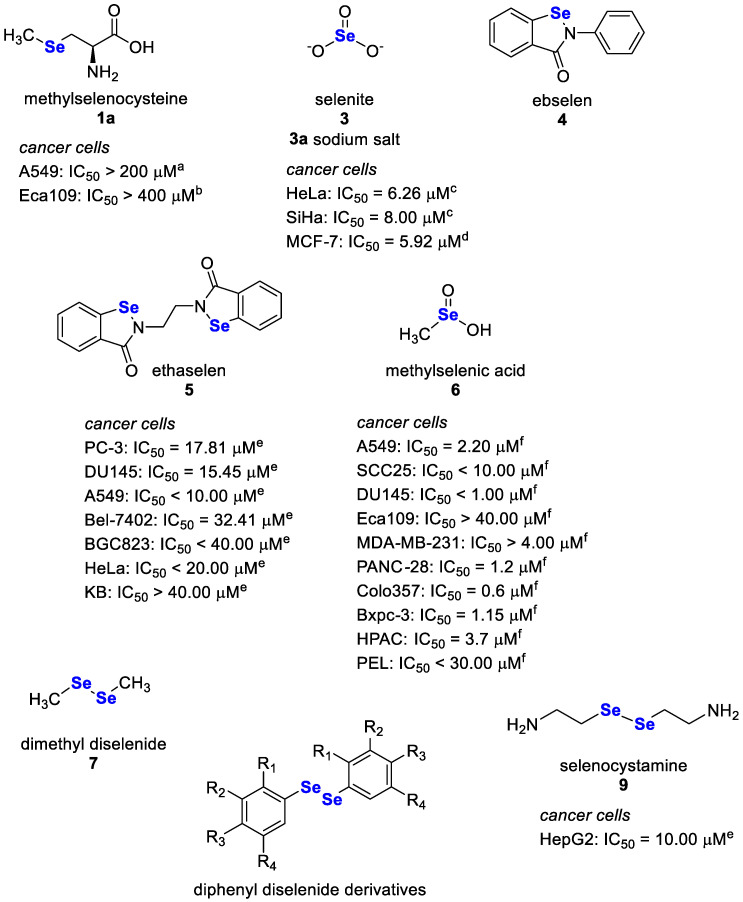

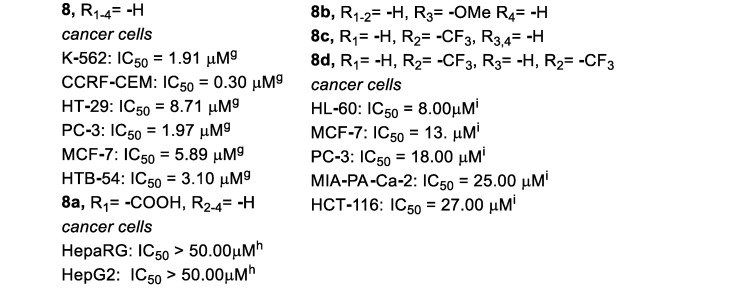
Chemical structures of representative inorganic and organic selenium compounds **1**-**8** discussed in this review. Data from: ^a^ [55], ^b^ [56], ^c^ [57], ^d^ [58], ^e^ [39], ^f^ [59], ^g^ [60], ^h^ [61], ^i^ [52].

Se-containing compounds can affect gene expression, cell signal pathways, DNA repair/damage, as well as angiogenesis and metastasis [52] through the formation of ROS and the oxidation of protein thiol groups. Evidence revealed that at low levels, these compounds induce antioxidant effects when selenium is incorporated into SePs, while pro-oxidant and anticancer effects occur at high doses [52]. Some compounds have also been found endowed with chemosensitising properties, potentiating the efficacy of anticancer drugs [53], likely through the inhibition of the efflux pumps [54]. Details about the most investigated compounds (Figure 4) and the significant findings collected in the last five years about their anticancer properties and efficacy are described in the following sections.

### 3.1. Selenites and Sodium Selenite

Selenite **3** and its sodium salt, sodium selenite **3a**, represent the most investigated inorganic Se-containing compounds. These molecules have been shown to possess anticancer activity in several cancer cell lines, including prostate, breast, lung, hepatoma, bladder, and osteosarcoma [52]. Recently, the anticancer effects of sodium selenite **3a** were also displayed in cervical cancer cells with IC_50_ values ranging from 6.26 to 8.00 μM and in the relative xenograft model [57]. Moreover, the antiproliferative activity of sodium selenite **3a** was reported in breast cancer MCF-7 cells (IC_50_ = 5.92 μM) [58] and in drug-resistant pancreatic cancer [62]. 

Previous studies have already highlighted that resistant cells are more susceptible to selenite than sensitive ones due to their high metabolic activity and redox imbalance, which led them to be more vulnerable to redox-active selenium [62]. Induction of oxidative stress plays a central role in the antitumor effect of **3** and **3a**, responsible for the damage at the mitochondria and endoplasmic reticulum levels, which determine the triggering of apoptosis [52]. Other types of cell death are involved in selenite **3** anticancer activity; the induction of necrosis has been observed in breast cancer MCF-7 cells, autophagy and necrosis in chemoresistant human bladder cancer RT-112/D21 cells [52] and ferroptosis in human breast, prostate and glioma cells [63].

Combination experiments to evaluate the chemosensitising properties of selenite have been carried out as well. The compound synergistically increased the effectiveness of imatinib, cisplatin, ethaselen **5**, and auranofin against colorectal, breast, lung and ovarian cancer cells, respectively [52,64]. Moreover, it has been shown that the antiproliferative activity of 5-fluorouracil (5-FU), oxaliplatin, and irinotecan in HCT116 colon cancer cells was increased by selenite **3** of about 1.1-fold, 2.7-fold, and 2.6-fold, respectively. Conversely, in SW620 colon cancer cells, 1.5-fold and 4.3-fold increases in 5-FU and oxaliplatin cytotoxicity were observed in combination with selenite [64,65]. Furthermore, it reduced the side effects of some cytostatic drugs, including 5-FU and docetaxel [64]. These interesting findings have allowed for the design of several clinical trials, most of which are ongoing.

### 3.2. Amino Acid-Derived Selenium Compounds

Selenocysteine **1**, methylselenocysteine **1a** and selenomethionine **2**, which contain a selenium atom in the place of sulphur, are the most studied selenoaminoacids as anticancer agents. Selenocysteine **1** is a naturally occurring amino acid carrying a Se–Se bond, whose oxidation leads to the generation of selenocysteine **1**; it is incorporated in SePs, such as GPx, which deal with cellular redox state maintenance [66], while selenomethionine **2** is unspecifically included into proteins instead of methionine amino acid and undergoes oxidative decomposition to its active metabolite methylseleninic acid **7** by an L-methioninase enzyme [67]. Available evidence highlighted that **1**, **1a** and **2** are endowed with antiproliferative activities mediated by an increased apoptotic rate in different cancer cells (IC_50_ > 100 µM) [52,68]. Selenocysteine **1** has been reported to induce cell death in different cell lines (e.g., melanoma, cervical, breast, liver, or lung cancer; IC_50_ values ranging from 3.6 to 37.0 µM) and in vivo xenograft models (10 mg/g body weight per day; ten-day treatment) [52]. It also impairs the viability of human hepatocellular carcinoma cells (IC_50_ > 80 µM) by inducing oxidative-mediated DNA damage [69]. Recently, it has been demonstrated to inhibit the viability of JEG-3 choriocarcinoma cells (IC_50_~20 µM) through the activation of oxidative stress, DNA damage, S-phase arrest and apoptosis [70]. In summary, this evidence suggests an interest in compound **1** with broad-spectrum anticancer activity and encourages further studies for confirmation.

Accordingly, Zhang et al. [71] highlighted that L-selenocysteine **1** decreased the viability of HepG2 cells (IC_50_~4 µM) and induced apoptosis. Korbut et al. [72] showed that selenomethionine **2** suppressed the growth of HT-29 colorectal cancer cells (IC_50_ values of 283 and 630 μM in HT-29 and HaCaT colorectal cancer cells, respectively) by affecting the Wnt/β-catenin cascade; moreover, the Se-containing compound induced cytotoxic effects at concentrations higher than 10 μM and affected the redox state of MCF-7 breast cancer cells, although at a lower extent than selenocysteine **1** [73]. 

Methylselenocysteine **1a** has been highlighted to affect the proliferation of A549 lung adenocarcinoma cells (IC_50_ > 200 μ), likely through promoting oxidative stress, despite low cytotoxicity in normal cells; moreover, it synergised the cytotoxic effects of different anticancer drugs [55]. It increased the etoposide cytotoxicity in HeLa cervical cancer cells by enhancing the gap junction activity through a GSH-dependent mechanism [74], and both methylselenocysteine **1a** and methylseleninic acid **7** induced cytotoxicity (IC_50_ > 400 μM), apoptosis and cell cycle arrest in the G0/G1 phase in oesophagus cancer cells [56]. 

The promising preclinical evidence led to the study of methylselenocysteine **1a** and selenomethionine **2** in clinical trials, especially as chemopreventive treatments, while no studies are reported for selenocysteine **1** [52]. Particularly, different trials explored the role of selenomethionine **2** as both chemopreventive and chemosensitising agents in prostate (NCT00736164, NCT00736645, NCT00030901, NCT00217516, NCT01497431, NCT00006392), colorectal (NCT00706121, NCT01211561, NCT00625183), lung (NCT00526890, NCT00008385), neck (NCT01682031) and thyroid (NCT04683575) cancers [75]. Likewise, methylselenocysteine **1a** has been assessed as a chemopreventive intervention for breast and prostate cancers (NCT01611038, NCT01497431) and as a chemosensitiser in patients with diffuse large B-cell lymphoma that has relapsed or not responded to treatment (NCT00829205) [75]. 

### 3.3. Methylseleninic Acid

Methylseleninic acid **6** is an organo-selenium compound obtained by the oxidative decomposition of methylselenocysteine **1a** [76]. Several in vitro studies demonstrated its chemopreventive and anticancer properties in different cancer cell lines, including prostate, head and neck, leukaemia, breast, lung, ovarian, pancreatic, and oesophageal squamous ones (IC_50_ values ranging from 1 to 40 µM) [59]; particularly, antiangiogenic, cell cycle blocking, and antiproliferative properties were reported [40]. Its anticancer effects were also displayed in a xenograft model of colon cancer [52]. The induction of oxidative stress, mediated by enhanced ROS generation and GSH depletion, seems to be the primary mechanism responsible for the anticancer effects of this Se-containing compound. 

Recently, the induction of entosis in pancreatic cancer cells by methylseleninic acid **6** has been also reported [77], and other studies have highlighted that this compound has modulated some cellular pathways (e.g., JAK-STAT3) involved in tumour initiation, proliferation, progression and metastasis [78,79]. Lastly, methylseleninic acid **6** has also shown chemosensitising properties, increasing the effectiveness of radiation and of several anticancer drugs (e.g., cisplatin, paclitaxel, etoposide, and doxorubicin) when used in combination [52,80,81]. Particularly, **6** was able to increase the proapoptotic effects of SN38, etoposide, or paclitaxel by about four-fold with respect to the drug alone, in prostate cancer DU145 cells [65]; the modulation of JNK-dependent molecular targets seemed responsible for these chemosensitising effects [52]. Recently, methylseleninic acid **6** has also been reported to sensitize head and neck squamous cell carcinoma (HNSCC) to radiotherapy by inducing lipid peroxidation [65]. Noteworthy, normal skin cells were not affected by it. Based on the present evidence, methylseleninic acid **6** can be considered a promising candidate for cancer treatment. 

### 3.4. Ebselen

Ebselen **4** is a synthetic Se-containing heterocyclic compound with chemopreventive properties due to its antioxidant, anti-inflammatory, and GPx-mimicking activities [4]. Many studies have investigated the pharmacological properties of compound **4**, highlighting its promising application for treating several disorders (e.g., bipolar disorders, hearing loss, malaria, and tuberculosis), among which include cancer [4,82]. Compound **4** exhibited antiproliferative properties: it increased the apoptosis process in human hepatoma and multiple myeloma cells through oxidative stress induction [52]. In HepG2 cells, 50 and 75 µM of ebselen **4** determined an increase in the apoptosis rate through the depletion of thiol-containing molecules [52], and 40 µM ebselen, for 4 h in human multiple myeloma cells, increased apoptosis. The antiproliferative effect was ascribed to the high increase in ROS levels, which determined the translocation of Bax to the mitochondria, the release of cytochrome c into the cytoplasm and the initiation of apoptosis [52]. A 58% reduction of tumour development with respect to the control group was observed in a pancreatic cancer xenograft model after treatment for 28 days with a dose of ebselen **4** equal to 160 µg [83]. Recently, it has also been reported as the first inhibitor of the m6A readers YTHDF proteins, which play a key role in regulating genes involved in pathological conditions [84].

Some studies have also highlighted that ebselen **4** increases the apoptosis rate of human breast cancer and glioblastoma cell lines when assessed in combination with γ-ray or TNF-α, respectively [39,52], and its ability to destabilise the 14-3-3 proteins in the SHSY5Y neuroblastoma cell line [85]. These proteins are overexpressed in several cancers and are associated with poor prognosis and aggressive tumour growth; therefore, targeting 14-3-3 proteins could be a new strategy by which ebselen **4** makes cancer cells more susceptible to chemotherapy treatment, thus overcoming drug resistance.

Currently, no clinical trials have been carried out to prove the efficacy of ebselen **4** in cancer therapy. However, some studies have tested its safety, tolerability, and pharmacokinetics in humans (NCT03013400, NCT01452607, and NCT02603081). Moreover, another trial (NCT number: NCT01451853, phase 2) has been designed to evaluate its efficacy and safety in preventing hearing loss induced by platinum-based cytostatic drugs (e.g., cisplatin, carboplatin) [75].

### 3.5. Ethaselen

Ethaselen is a close analogue of ebselen **4** and seems to be a promising molecule with potential anticancer properties. In vitro and in vivo experiments highlighted its antitumor properties against several cancer models, including lung, tongue, stomach, liver, colon, prostate, cervix, nasopharyngeal cavity, and leukaemia [39]. Particularly, ethaselen **5** showed cytotoxic effects in PC-3 and DU145 cell lines with IC_50_ values of 17.81 and 15.45 μM, respectively [39]. Moreover, it showed potent antiproliferative effects on A549, Bel-7402, BGC823, HeLa and KB cell lines, with IC_50_ values ranging approximately from 6 μM to 40 μM [39]. The inhibition of mammalian thioredoxin reductase (TrxR), which in turn blocks cell proliferation and induces apoptosis, seems to be the mechanism responsible for the anticancer effects of ethaselen [4,39,52]. 

In addition, it has been highlighted that ethaselen **5** can synergistically increase the efficacy of chemotherapeutic drugs. It increases the sensitivity of leukemic-resistant and colon cancer cells to cisplatin and sunitinib treatment by about 20- and 2-fold, respectively [39,86]; similarly, it enhanced cisplatin toxicity in lung xenografts models [83]. Lastly, ethaselen **5** sensitised non-small cell lung cancer cells to radiotherapy [53]. At present, **5** is in a phase 1c clinical trial (NCT02166242) for treating patients with non-small cell lung cancer (NSCLC) overexpressing TrxR [75]. 

### 3.6. Diselenides 

Diselenides are organic selenium compounds containing a Se–Se group with a low bond energy, which makes the compound easy to oxidize or reduce [87]. Some compounds, such as dimethyl diselenide **7** and diphenyl diselenide **8**, have been found to possess antioxidant properties, although others (e.g., dipropyl diselenide) exhibited pro-oxidant effects [52]. Diselenides, selenocystamine **9** (IC_50_ > 10 µM in HepG2 cells) and diphenyl diselenide **8** (IC_50_~15–30 µM) have promising anticancer activities [39,51]; along with the antiproliferative properties in different cancer cells, likely mediated by the apoptosis pathway [52]. Diphenyl diselenide **8** was mainly studied for its antioxidant and genoprotective properties in combination with conventional anticancer drugs [52,88]. However, it showed a toxicity risk in vivo, depending on the route of administration and dosage; therefore, low toxic derivatives have been developed [52,89]. Among the diselenides, a diarylseleno derivative from diphenyl diselenide **8** was found to be cytotoxic against different human cancer cell lines (IC_50_ values ranging from 0.04 to 30 μM), especially in acute lymphoblastic leukaemia CCRF-CEM cells [60]; similarly, 4,4′-dimethoxy- (**8b**) and 3,3′-ditrifluoromethyl- (**8c**) diphenyl diselenide induced apoptosis and cell cycle arrest via a caspase-dependent pathway at 20 μM, while 3′,5′,3,5-tetratrifluoromethyl-diphenyl diselenide **8d** exhibited marked cytotoxicity in vitro in different cancer cell lines (i.e., HL-60, MCF-7, PC-3, MIA-PA-Ca-2 and HCT-116; IC_50_~8–30 μM) [52]. 

Lastly, DSBA (2,2′-diselenyldibenzoicacid) **8a** was found to retain the in vitro pharmacological properties of diphenyl diselenide but with lower toxicity (IC_50_ > 50 μM); it also produced chemopreventive properties in vivo by preventing the suppression of bone marrow hematopoietic cells induced by ionising radiation exposure: this effect was ascribed to the activation of the Nrf2 pathway [61]. Altogether, the present findings suggest an interest in diphenyl diselenide nontoxic derivatives as anticancer agents, although further studies and clinical trials are required to confirm their effectiveness. 

### 3.7. Polysaccharide-Derived Selenium Compounds

Selenium polysaccharides are natural compounds generated by the combination of inorganic selenium and polysaccharides and are expected to join the properties of both molecules. Some compounds have been isolated from nature and evaluated for their antiproliferative properties in different cancer models. Remarkably, the selenium-polysaccharide Se-GFP-22, isolated from the fungus *Grifola frondosa* (Fr.) S.F. Gray, was shown to counteract the immunosuppression induced by cyclophosphamide in mice, likely boosting the immune system by the upregulation of MAPKs signalling [90]. This suggested a possible role of Se-GFP-22 as an adjuvant strategy to relieve chemotherapy-induced immunosuppression. 

Moreover, the selenium polysaccharide fraction Se-POP-3, obtained from *Pleurotus ostreatus* (Jacq.) Kummr, was found to moderately affect the viability of human gastric cancer MGC-803, colon cancer HCT-116 cells, and normal intestinal epithelial NCM460 cells (IC_50_ > > 600 μg/mL), to induce apoptosis and to block cell migration by disrupting the Bax/Bcl-2 ratio and by inhibiting the epithelial-to-mesenchymal transition (EMT); it is noteworthy that Se-POP-3 did not affect the growth of normal cells [91,92]. This evidence suggested a possible interest in Se-POP-3 as a novel anticancer strategy; however, further studies are required to confirm the antitumor effects in vivo and in clinical trials. 

### 3.8. Selol

This compound was obtained by reacting triglycerides from sunflower oil with selenic acid; however, the precise structure is not yet known [93]. The activity of selol is strictly dependent on its Se content, based on the number of dioxaselenolane rings in the molecule. While selol 2% has been reported to exert antioxidant activity, selol 7% showed cytotoxic effects [52]. The compound was found to inhibit cell proliferation and induce apoptosis in leukemic cells, being more effective in resistant ones. The following IC_50_ values were highlighted: 25 µg Se/mL in HL-60, 20 µg Se/mL in vincristine-resistant HL-60/Vinc, and 15 µg Se/mL in doxorubicin-resistant HL-60/Dox cells [94]. Similar effects were also observed in androgen-dependent prostate cancer cells but not in normal cells [52].

The chemosensitising properties of selol have been highlighted since it increased the doxorubicin antiproliferative effects by reducing the expression of MYC and BCL2 genes [95]; moreover, it enhanced the analgesic effects of fentanyl, buprenorphine and morphine in an animal model of vincristine-induced hyperalgesia [52]. The pro-oxidant effect of selol and its ability to inhibit TrxR could be involved [52,96]; however, the precise mechanism and the molecular targets of selol still have to be determined. 

### 3.9. Selenium in Hybrid Compounds 

**Figure 5:**
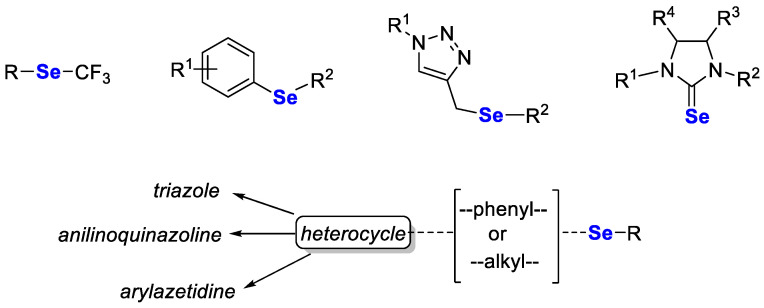
General structures of selenium–based hybrids described in the review of Singh et al. [97].

As cancer is a complex disease, hybrid compounds that act on several biological targets simultaneously may be more effective in treatment than selective drugs. Recently, Singh et al. [97] reviewed such compounds, of which the general structures are shown in Figure 5.

**Figure 6:**
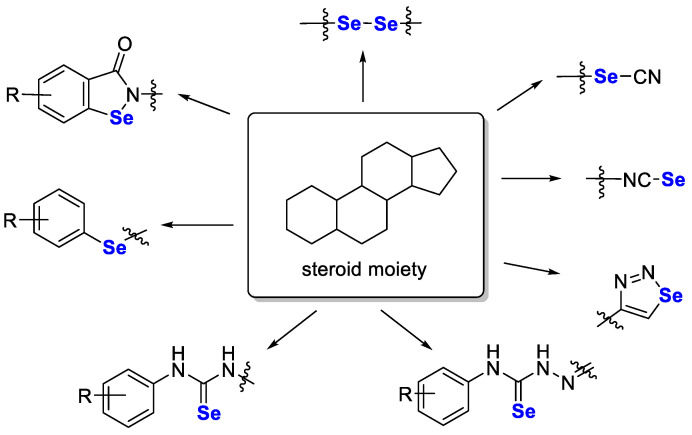
General structure of selenium-based hybrids of steroid derivatives described in the review of Jastrzębska et al. [98].

Similarly, a fascinating review paper on selenosteroid compounds was recently described by Jastrzębska et al. [98]. In these compounds, selenium (in an organoselenium moiety) was attached directly to the steroid molecule or by linking to a selenium-containing group (i.e., selenourea, selenocyanine or isoselenocyanine moiety). Such compounds have shown promising antioxidant and/or anticancer activity in in vitro studies. The general structure of such compounds with possible modifications is shown in Figure 6.

Here, we focus on hybrid selenium compounds that have not been described in earlier mentioned publications and discuss their anticancer activity.

#### 3.9.1. Isocombretastatin A and Phenstatin-Based Hybrids

**Figure 7:**
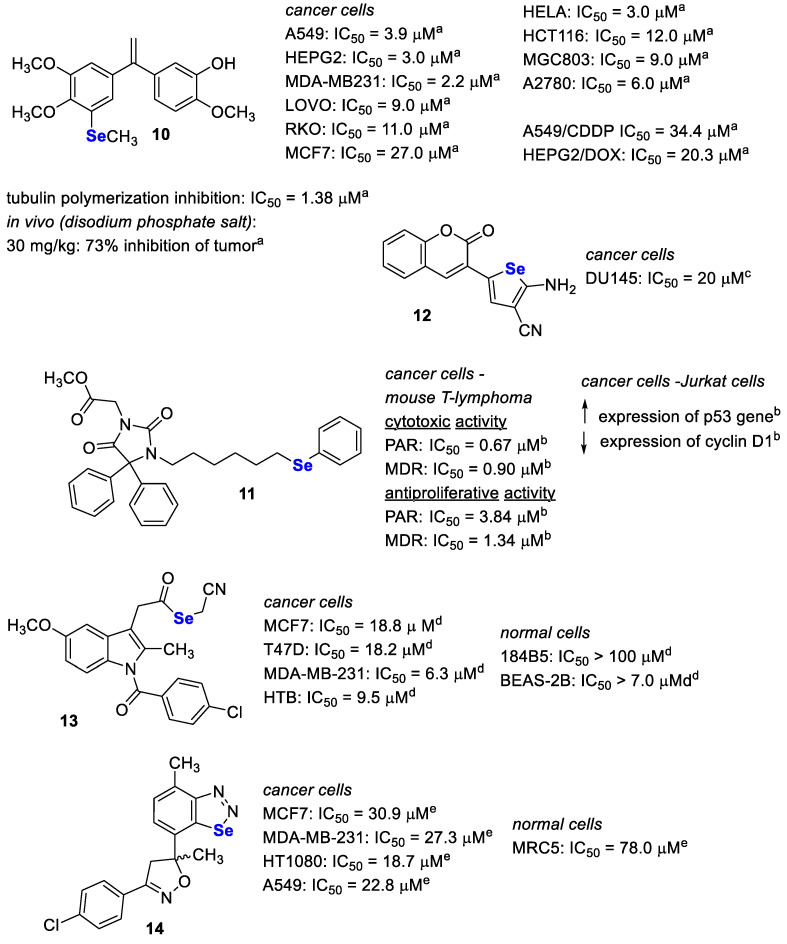
Structures and activity of the most potent selenium-based hybrids **10**-**14**. Data from: ^a^ [99], ^b^ [102], ^c^ [105], ^d^ [106], ^e^ [109].

Pang et al. [99] described hybrid compounds formed by combining methyl(phenyl)selane with tubulin polymerisation inhibitors (isocombretastatin A and phenstatin) in a single molecule. Antitubulin compounds are drugs used in the treatment of solid tumours, as well as in haematological malignancies such as lymphomas [100,101]. Thus, such dual-targeting compounds with synergic effects could show higher therapeutic efficacy in cancer. Pang et al. synthesised 20 compounds and tested them in cancer cell lines (A549, MDAMB231, HEPG2, LOVO and RKO) for antiproliferative activity after 48 h. Most compounds were active in the nanomolar range (<1000 nM) in all the tested cells. The most active compound, **10** (Figure 7), caused proliferation block with IC_50_ values in the range from 2.2 nM (in MDA-MB-231 cells) to 11 nM (in RKO cells). This compound, further tested on other cancer cell lines (MCF7, HELA, HCT116, MGC803 and A2780), also exhibited auspicious activity (IC_50_ = 3–27 nM). Moreover, **10** was tested in the cisplatin-resistant (A549/CDDP) and the doxorubicin-resistant (HEPG2/DOX) cell lines and showed antiproliferative activity with IC_50_ values of 34.4 and 20.3 nM and resistance factor values of 8.8 and 6.8, respectively. Additional studies confirmed the ability of **10** to inhibit tubulin polymerisation with an IC_50_ of 1.38 nM. Furthermore, in vitro efficacy of **10** was confirmed later by in vivo studies in the A549 xenograft model in nude mice. To enhance biodegradability, disodium phosphate salt of **10** was used as a prodrug for this study. The compound was administered intraperitoneally at a dose of 30 mg/kg body weight for the tested period, and a 73% reduction in tumour weight was observed in comparison to the control group.

#### 3.9.2. Phenylselenoethers

Ali et al. [102] described 13 phenylselenoethers as P-glycoprotein (ABCB1) MDR efflux pump modulators. The hybrids were obtained by attaching a phenyl ring via a selenoether bond with aryl-hydantoins (three compounds), (aryl)piperazines (six compounds) and 1,3,5-triazines (four compounds). Cytotoxic and antiproliferative activity of compounds were evaluated in the resistant (MDR) and in the sensitive (PAR) mouse T-lymphoma cells. The highest cytotoxic effect was shown for compound **11** (Figure 7) with IC_50_ of 0.67 µM for PAR and IC_50_ of 0.90 µM for MDR. This compound also exhibited an interesting antiproliferative effect in tested cell lines (IC_50_ = 3.84 µM for PAR; IC_50_ = 1.34 µM for MDR). In further tests, in JURKAT leukaemia cells, the effect of compound **11** alone or in combination with the chemotherapeutic doxorubicin was evaluated after 24 and 72 h, indicating a positive effect of co-treatment of compound **11** with doxorubicin against cell proliferation, especially after 72 h. Next, it was demonstrated that anticancer activity relates to the ability of compound **11** to inhibit the expression of cyclin D1 and induce the p53 protein expression level.

#### 3.9.3. Coumarin-Based Hybrids

The coumarin ring is present in several compounds that have shown a variety of anticancer activities (e.g., inhibition of angiogenesis), kinases or enzymes (e.g., telomerase, aromatase, carbonic anhydrase or steroid sulfatase) [103,104]. Yildirim et al. [105] described a small series (four compounds) of coumarin hybrids with 2-aminoselenophene-3-carbonitrile (Figure 7). The compounds were tested against prostate cancer on DU145 cells and showed antiproliferative activity with IC_50_ ranging from 20 to 44.0 µM (higher than for the corresponding coumarin analogues). The highest activity was observed for compound **12** (IC_50_ = 20 µM; Figure 7). Next, an attempt was made to clarify which apoptosis pathway was affected by the tested compound (at 20 µM concentration) by measuring the levels of caspases 3, 8 and 9 in DU145 cells by ELISA. The results showed that compound **12** caused an increase in the expression of the tested caspases, with caspase 8 being not statistically significant.

#### 3.9.4. Non-Steroidal Anti-Inflammatory Drug-Based Hybrids

Ramos-Inza et al. [106] attached the selenoester moiety to known non-steroidal anti-inflammatory drugs (NSAIDs) such as aspirin, salicylic acid, naproxen, indomethacin and ketoprofen. Such drugs (especially aspirin) might have a beneficial effect on breast cancer patients, acting as a prophylactic or reducing mortality [107,108]. The synthesised 25 compounds were preliminarily tested for cytotoxic effects in the MTT assay in a panel of four cancer lines (HTB54, HT29, DU145 and MCF7) and two non-cancer lines (184B5 and BEAS-2B). The selected five most active compounds were then tested in other cancer lines (T47D, MDA-MB-231, H1299) and in a panel of 60 lines in the NCI Development Therapeutics Program (US). These studies showed that two compounds, derivatives of indomethacin, had very strong cytotoxic effects, especially in breast cancer cells. Inhibition of cancer cell proliferation by these compounds was further confirmed in the Trypan Blue assay in MCF7 and MDA-MB-231 cells. In addition, Annexin V, Cell death and Caspase3/7 assays were performed to measure cell death by apoptosis and showed that compound **13** (Figure 7) effectively induced apoptosis and increased caspase 3/7 activity.

#### 3.9.5. Isoxazole-Based Hybrids

Five benzo[1,2,3]selenodiazole hybrids with an isoxazole moiety were obtained by Oubella et al. [109]. Isoxazole derivatives (e.g., *luminespid*) [110] are described as potent anticancer compounds [111]; thus, combining this moiety with selenium-containing derivatives may allow for compounds with greater anticancer activity. The final five benzoselenadizole-isoxazoles were estimated by MTT assay on four tumour lines (HT1080, A549, MCF-7 and MDA-MB-231) [109]. Results (after 24 h) showed lower cytotoxic activity of these compounds (IC_50_ from 18.7 to >100 µM) in comparison with their semicarbazone precursors (IC_50_ from 10.9 to 21.6 µM) with the most promising compound in this series represented by **14** (Figure 7). The test on healthy cells (MRC5) showed their moderate survival after **14** treatment; furthermore, the study of apoptosis induction by **14** in HT1080 and A549 cell lines in the Annexin-V binding assay showed a 7- and 17-fold increase in apoptosis, respectively, compared to control cells. Further studies confirmed that this process occurs through caspase 3/7 induction.

## 4. Role of Selenium in Inflammation and Immunity

Selenoproteins regulate the health of many organs by maintaining broader homeostasis through regulation of the thyroid hormone level, endoplasmic reticulum stress and antioxidant defence [7], but also immune and inflammatory responses. Many members of the selenoprotein family function as enzymes related to immune functions. The most important are GPXs, thioredoxin reductases (TXNRDs), iodothyronine deiodinases (DIOs), methionine-*R*-sulfoxide reductase B1 (MSRB1), and selenophosphate synthetase 2 (SPS2).

The concept of linking inflammation and cancer was extensively developed, and today, we have a reasonably broad understanding of processes that occur in microenvironments rich in inflammatory cells, which produce pro-survival and pro-migration factors, thus promoting cancer development and metastasis. Consideration of the role of selenium compounds in the inflammatory process is most warranted. It is well known that the balance of REDOX is regulated by ROS, which, being both toxic and signalling molecules, play an essential role in the inflammatory process [112]. In line with this, the knowledge of the effect of selenium on the immune system has dramatically improved, and at present, selenium is considered a micronutrient belonging to the immunoceuticals, which are immune-modulating compounds that tune up the immune system.

### 4.1. Cytokines and Inflammatory Signals

Cytokines affect the growth of all blood cells and other cells that help the body’s immune and inflammation responses. They also help increase the anti-cancer activity by sending signals that help to kill abnormal cells as reported in a recent paper, indicating that TNF-α and IFN-γ together induce cell death in 13 distinct human cancer cell lines derived from colon and lung cancer, melanoma, and leukaemia [113] and enable normal cells to live longer. Epidemiological studies have shown a relationship between the level of selenium and the risk of cancer in people with low levels of selenium [114]; additional Se supplementation might result in upregulating the key pro-inflammatory signals. In the anti-tumour effect, B-cells have also contributed as cells presenting antigens which help tumour-infiltrating T cells to proliferate [115] while natural killer cells (NK) carry out immune surveillance via different receptors on their surface which can programme cell death in cancer cells without major histocompatibility class I [116].

**Figure 8:**
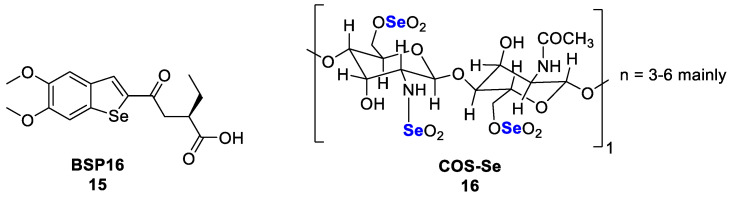
Se-containing compounds **15** and **16** with an immunomodulatory effect in cancer [118,120].

Recently, an attractive approach in anticancer immunity is the activation of the stimulator of the interferon genes pathway (STING). STING is a protein expressed in the endoplasmic reticulum, mainly in immune cells. It is activated by DNA released from phagocyted tumour cells and produces the necessary cytokines to induce strong anti-tumour T-cell reactions [117]. BSP16 (**15**, LF250) (Figure 8), a selenium-containing STING agonist, was reported as an antitumor agent that caused tumour regression and induced antitumor immunity. BSP16 **15** (0.1–100 μM) can selectively stimulate the STING pathway in human leukaemia monocytic cell line (ISG-THP1) and mouse macrophage cell line (ISGRAW264.7) with EC_50_ values of 9.24 and 5.71 μM, respectively. In an in vivo study, BSP16 **15** showed an excellent pharmacokinetic profile as an oral drug (oral, 15 and 30 mg/kg, q3d; oral, 20 mg/kg, q5d) with regression of tumour after 21 days in a colon carcinoma tumour model [118]. This direct action on STING is crucial when mutagen-induced inflammation-driven epithelial cancer is developed. Another interesting study demonstrated that Se-enriched mushroom extract (SME) regulated the expression profile of the cancer cell proliferation factor Raf-1 and of the pro-apoptotic-related factors P53 and caspase-3 as well as the production of inflammatory cytokines IL-6 and IL-10 [119]. Dietary consumption of selenium to prevent cancer development is a promising approach. The efficient delivery of selenium is crucial in this case, which is why improved forms of organic selenium are developed. Chitosan oligosaccharide-conjugated selenium (COS-Se, **16**, Figure 8) is a new chemical structure similar to natural selenium oligosaccharide. In vitro studies demonstrated that COS-Se **16** increased immunologic mediators such as TNF-α and IL-1β in peritoneal macrophages at concentrations of 50, 100, 200, and 500 μg/mL. Equally interesting results described in this publication relate to in vivo studies showing that COS-Se **16** supplementation at doses of 50 and 100 mg/kg effectively inhibited the growth of transplanted gastric adenocarcinoma in Balb/c nu mice with inhibitory rates of 29% and 33%, respectively. Two mechanisms were behind this tumour suppression: the reduction of MMP-9 and VEGF serum levels in mice, which means that tumour metastasis and angiogenesis were blocked [120].

An inorganic form of selenium, such as selenium selenite **3**, has also been shown to decrease oxidative stress in cancer tissues [121]. Adiponectin knock-out mice were under a Se-enhanced diet, which contained 0.75 ppm (200 μg per day) of added Se for 194 days; chronic inflammation-induced colon cancer (CICC) was initiated with the colon cancer agent 1,2 dimethyl hydrazine (DMH) along with dextran sodium sulphate (DSS). The results presented by Saxena et al. demonstrated a significant reduction in tumour number and in common clinical symptoms of colon cancer. The oxidation products from proteins and lipids, nitrotyrosine and 4-hydroxynonenal, respectively, were also significantly decreased in colon cancer tissue of knockout mice. DSS + DMH administered animals on a Se-enriched diet presented a lower degree of inflammation, immune cell infiltration and tumour growth compared with the same animal model on a regular diet.

### 4.2. Selenoproteins and Inflammation

Selenium is the integral compound of selenoproteins in the human body. In this review, we briefly introduced some selenoproteins and their role in inflammation. SeP is mainly synthesised in the liver and, in human plasma, is the main selenium-containing protein. It is unique since it has as many as 132 selenocysteines in its molecule, making it energetically costly to produce; however, it supplies tissues (such as the brain, kidney and testis) with selenium [122]. SeP also serves in antioxidant protection, which explains the fact that its reduced expression is observed in several types of tumours, including prostate, renal, hepatocellular or colorectal cancer [123]. Particularly noteworthy is the fact that during tumour development, a certain activity of the cells of the immune system is observed. It is also noted that *SELENOP* mRNA expression might be related to some immune functions, as it was positively correlated with immune marker genes for macrophage subtypes, including tumour-associated macrophages (*CD80, CD86* and *HLA-G*), M1 macrophages (*CD36, IL-6* and *NOS2*), and M2 macrophages (*TGFβ, STAT6* and *IL-10*) in the following cancers: kidney clear cell carcinoma, brain lower grade glioma, lung adenocarcinoma and skin cutaneous melanoma [123]. SeP also reflects the selenium nutritional status in brain tissues, where it is the only selenium donor. It was shown that selenium supplementation in BTBR mice significantly decreased the mRNA and protein levels of inflammatory cytokines IL-6, IL-1β and TNF-α in the hippocampal tissue, pointing out the protective role of selenium [124].

### 4.3. Selenium Deficiency Impaired Immune Function

Studies have found that selenium deficiency might impair immune function and lead to inflammation-related diseases. Selenium deficiency blocked the glutathione and thioredoxin antioxidant systems and led to redox imbalance. The activation of NF-κB and HIF-1α transcription factors are observed, which results in increasing of pro-inflammatory cytokines (IL-1β, IL-6, IL-8, IL-17, and TNF-α), decreasing anti-inflammatory cytokines (IL-10, IL-13, and TGF-β) and increased expression of the downstream genes COX-2 and iNOS, which in turn induced inflammation [125]. Other structural studies using transmission electron microscopy revealed damages in the jejunum epithelial microvilli of broilers, mitochondrial swelling and vacuolation due to the lack of selenium. In the same study at the molecular level, intestinal inflammatory factors (NF-κB P65, IKKα, IKKβ, IL-1β, IL-6, IFN-γ, and TNF-α) were upregulated, while anti-inflammatory IL-10 was significantly downregulated [126]. Only a few studies focused on the epithelial barrier integrity following enteric infection; in a paper on this issue, the mice fed with a selenium-supplemented or a selenium-deficient diet were infected with *C. rodentium* strain ICC 169 (nalidixic acid resistant), and the analysis of ILC3 and Th17 cells was performed. In the group of mice with selenium supplementation, this cell population was much higher compared to the selenium-deficient group, which would suggest a greater capacity for the clearance of the enteric pathogen and for maintaining the epithelial barrier integrity of the colon during infection. Moreover, if mice fed with the selenium diet were treated with CAY10397 (inhibitor of 15-hydroxy prostaglandin dehydrogenase), this treatment diminished the protective effect of selenium, suggesting that the oxidation of lipid mediator PGE_2_ is a key protective mechanism that is associated with selenium levels [127].

### 4.4. Selenium on Immune Cells Activity

**Figure 9:**
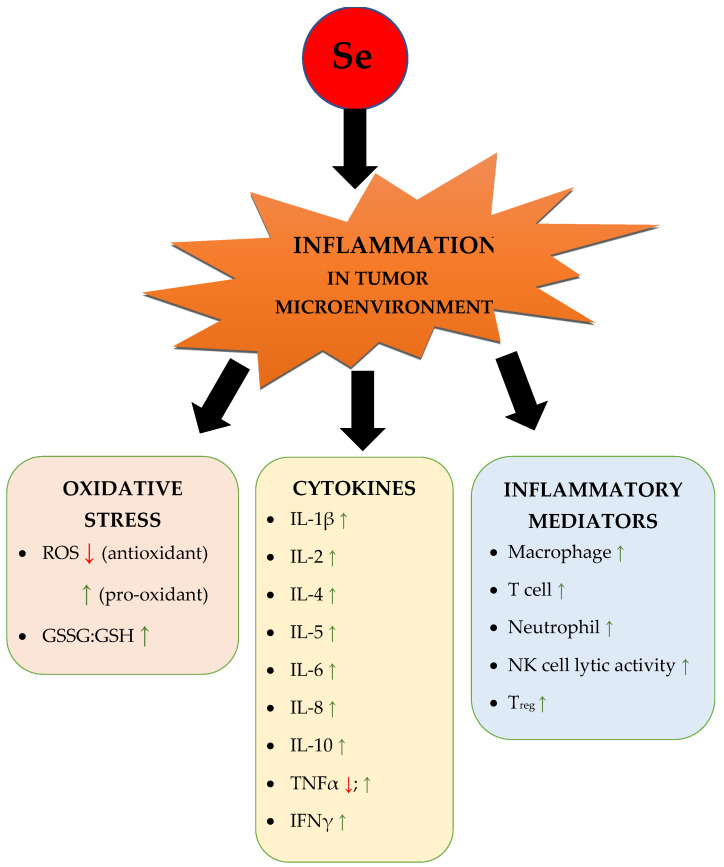
Overview of the influence of Se in the inflammatory tumour microenvironment. A green upward-facing arrow (↑) indicates that Se has a stimulating effect, while a red downward-facing arrow (↓) indicates that Se has an inhibiting effect. Abbreviations: ROS—reactive oxygen species; GSSG:GSH—the ratio of oxidized to reduced glutathione; IL—interleukin; TNFα—tumour necrosis factor alpha; IFNγ—interferon gamma; NK—natural killer [52,119,120,123,132,133,134,135,136,137,138,139,140,141,142,143,144,145,146,147,148,149,150,151,152].

The reported therapeutic role of Selenium in stimulating immune response is due to its ability to target different populations of immune cells (Figure 9).

It has been reported that the knockdown of selenoproteins significantly reduces the number of mature T-cells in lymphoid tissues [128]; for this reason, a significant change in tissue inflammation is observed.

Concerning T cells, it has been reported that knockout for the gene TSRP has an important readout in immunomodulatory effects. TSRP is a gene involved in the synthesis of selenoproteins affecting not only T-cell proliferation, differentiation and activation but also in controlling the number of T-helper cells with a substantial impact on B-cell activity and immunoglobulin production [128].

In Crohn’s disease, a well-characterized chronic inflammatory intestinal disease, it was observed that there is a deficiency in some metabolites, including selenium. Notably, the supplementation of selenium reduces the symptoms of this disease and regulates the differentiation of Th1 cells; this happens through the involvement of selenoprotein W-mediated (SELW-mediated) oxygen-reacting species scavenging [129].

Concerning cancer, it has been proven that Selenium is able to increase the proliferation and activation of CD8-positive cells, a population of immune cells found able to fight tumours in preclinical and clinical studies [129].

Molecularly, CD8+ cytotoxic T-cells play an important role in anticancer immune response since they are able to recognize cancer cells and induce cell death via the induction of the apoptotic pathway [129].

In addition, macrophage activity is regulated by selenium presence in the serum; this reflects a significant impact on the inflammatory signalling and the anti-pathogen response efficiency. Specifically, it seems that selenium was able to induce the switch of macrophages from the M1 pro-inflammatory phenotype to the M2 anti-inflammatory phenotype [130], regulating macrophage phagocytosis and migration properties.

Concerning neutrophils, endogenous oxidative stress is defeated by selenium supplementation [131] while in natural killer cells, selenium supplementation relates to NK activation and to a consequent increase in TNFα and IFNγ secretion and an augmentation of lytic activity.

### 4.5. Selenium and Selenium-Containing Compounds Enhance Cancer Immunotherapy

Concerning cancer immunotherapy, selenium has been proposed and validated as a potent agent. It has been proven that the selenoprotein SELENBP1 in colorectal cancer (CRC) has a lower expression compared to normal conditions. Notably, its expression is strongly correlated to the activation of immune cells and to the release of chemokines, and thus, it plays a central role and strongly influences immune infiltration in this kind of cancer [153].

Moreover, there are some initial observations about the cytotoxicity of selenium-immunoconjugates against triple-negative breast cancer [154]. In this work, IgG1 antibodies Bevacizumab and Trastuzumab were conjugated with the redox form of selenium in order to form Selenobevacizumab and Selenotrastuzumab and were tested in different cell lines with a strong arrest of cancer cells growth.

### 4.6. Selenium on Infection Diseases and Vaccination

Due to the pleiotropic effects that selenium can have on cellular pathways and cellular responses, it is quite challenging to circumscribe the individual molecular mechanism through which selenium is able to potentiate cellular immunity. However, there are some theories based on specific selenoproteins to take into consideration. One of these proteins is represented by the selenoprotein MSRB1, which is able to regulate actin polymerization, promoting phagocytosis and cytokine secretion [155]. Another important factor is the selenoprotein SELENOK, which is able to activate calcium reflux during immune cell activation [156].

Selenium supplementation is also able to enhance GPX4 in T-cells. In addition to the previously described effect of selenium in regulating GPX4, the major lipid peroxidase scavenger, its selenium-dependent increase enhances follicular helper T-cells (T_fh_) number, viability and antibody response in vivo in immunized mice after influenza vaccination. Moreover, the Selenium–GPX4 axis protects follicular helper T-cells from ferroptosis [157].

Besides the role of selenium and selenoprotein in inducing an immune response in cancer contexts, they can also directly affect viruses’ proliferation and infection. In particular, it has been described that the Coxsackie virus shows higher toxicity when the selenium level is low [158]. The low selenium level can cause an oxidative condition through which oxidative pressure can enhance the probability of accumulating mutations, allowing for viral evolution [159].

Other observations indicate that selenium levels decrease with the development of AIDS [160], and some selenoproteins such as GPX1, GPX4, and TRX1 decrease with HIV infection [161]; notably, TRX1 selenoprotein can inhibit in vitro HIV replication targeting Tat protein [162].

In addition, vaccine efficacy seems to be improved with the supplementation of selenium; a selenium-based approach against poliovirus infection has been proposed and validated; it seems that the vaccine’s efficacy against this specific infectious agent is significantly improved when it is administrated with selenium [163].

Selenium deficiency in the diet is also correlated to COVID-19 mortality. The selenium levels in patients who survived COVID-19 were significantly higher than those observed in dead patients [164]. Selenium shows an antiviral effect due to the regulation of CD4+ response through stimulation of proliferation, differentiation and activation of these cells; moreover, it is crucial for the cytotoxic effect of CD8+ cells and natural killer cells (NK) [165].

Finally, in recent years, great interest has been given to selenium nanoparticles (better described before) as a powerful strategy for cancer immunotherapy [133,134].

In a study, the efficacy of a selenium (Se)-bearing ruthenium (Ru) complex (RuSe) for immune-sensitising natural killer cells for immunotherapy purposes against prostate cancer was demonstrated [132]. Pre-treatment of prostate cancer cells with RuSe potentiated the lysis potency of NK cells and increased the release of IFNγ and IL-2 [132]. IFNγ, in turn, has cytostatic and pro-apoptotic functions and is useful for adjuvant immunotherapy for different types of cancer, as it can stimulate an anti-tumour immune response.

## 5. Selenium-Containing Nanoparticles (SeNPs) as An Innovative Therapeutic Strategy

Nanomaterials represent a heterogeneous family of particles, including liposomes, micelles and organic or inorganic nanoparticles whose potential roles as anticancer drugs or small molecules or RNA therapeutics carriers have been recently proposed to enhance their effective delivery in target cells [166,167]. The interest in nanomaterials grew in biomedicines due to the advantages related to their use; specifically, they are characterised by solubility and stability, they can be engineered to increase their specificity to target cells, and they show high biocompatibility, a low grade of immunogenicity and low side effects [167].

**Figure 10:**
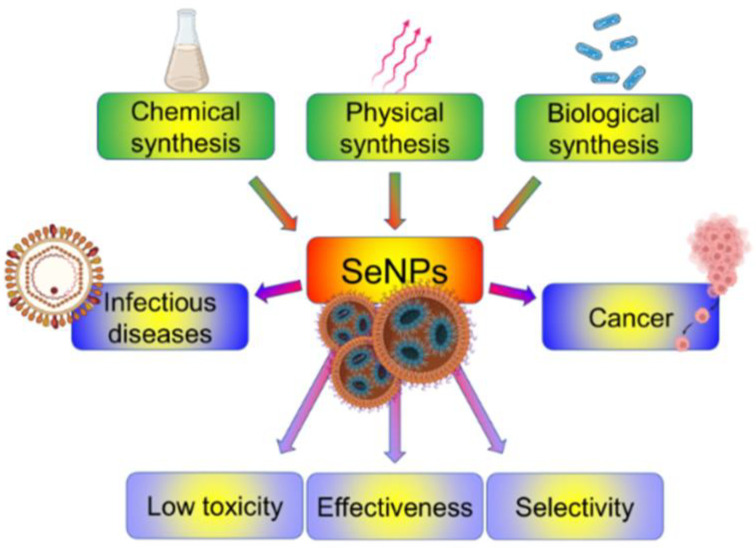
Overview of the properties and effects of SeNPs; in green are highlighted the SeNPs synthesis approaches, in blue their target diseases and in purple the advantages of SeNPs-based therapies. Relative references are reported in the text.

SeNPs show numerous advantages, such as high degradability, low toxicity and anticancer, antimicrobial and antiviral activities [168]. With respect to the low toxicity, it has been widely reported that this selectively protects normal cells from the cytotoxic effects of the treatment against cancer [169,170]; moreover, SeNPs can be rapidly excreted without long-term toxicity [171]. Their efficacy has been reported against oxidative stress and inflammation-mediated disorders (e.g., cancer, arthritis, diabetes and nephropathy [172]). These particles are also capable of targeting macrophages to activate innate immunity for antimicrobial inhibition through the production of cytokines [173], and these abilities have been translated into the field of cancer treatment since these nanoparticles can regulate tumour-associated macrophages and T cells (Figure 10) [140].

### 5.1. Preparation of SeNPs

The preparation of SeNPs can be performed through different approaches. The chemical reduction method (using vitamin C, sodium sulphite, sodium thiosulfate and hydrazine as reducing agents) is the most convenient, but other strategies have been developed. Hydrothermal synthesis is cheap, simple, efficient and produces functional nanoparticles with different shapes and diameters [174]. However, this preparation strategy requires high pressure and temperature in a specific hydrothermal autoclave reactor to produce small particles, SeNPs can show some agglomeration due to high surface energy and electrochemical properties; the process is difficult to control, and there is a low reliability and reproducibility [175,176]. Moreover, SeNPs can be formed using some chemical templates (e.g., PEG200, SPS, folate, hyaluronic acid, PEI) as stabilisers. Another approach to produce SeNPs is laser irradiation, also known as pulsed laser ablation (PLA) or deposition, exploited to inhibit bacterial growth [177]. Finally, in light of the so-called green chemistry, a large spectrum of plants, bacteria, and fungi have been reported as able to synthesise selenium-containing highly stable nanoparticles based on their ability to reduce selenite [178]. This last approach is highly controllable [179], produces highly pure SeNPs, displays great potential for anti-bacterial and anti-cancer treatments and is extremely cheap and fast, non-toxic, and overall, it is completely biocompatible.

Based on the oral administration of NPs and in order to limit the reduced selenium absorption and to improve the availability of Se, the production of SeNPs was implemented using the Eudragit^®^ RL and Eudragit^®^ RS polymers [180]. Recently, a new method for the production of SeNPs with high stability, good biocompatibility and narrow size that is environmentally friendly and economical has been proposed. This method uses the yeast as a bio-reducing agent, and the derived nanoparticles are stable and have fewer side effects in mice when added to their feed, protecting the liver, spleen, and kidney from oxidative stress, stimulating the humoral immune potential and significantly increasing the levels of IgM, IgA, and IgG [181], thus representing a potential substitute for antibiotics.

### 5.2. Therapeutic Potential of SeNPs as Drug Carriers

Moreover, since nanomaterials can be synthesised in order to contain selected elements [182], the incorporation of selenium into polymers has been described as a good strategy for the delivery of drugs, warranting low toxicity if used at low doses (less than 50 mg for adult people) and sensitivity to oxidative/reductive and radiation stimuli. Since redox conditions significantly differ between cancer and non-cancer cells, selenium-containing nanomaterials can be considered good candidates for the delivery of anticancer drugs. Moreover, SeNPs induce cell cycle arrest at the S phase [183] by deregulating the eIF3 protein complex [184] with a high selectivity against cancer cells (higher than selenium Se + IV at similar concentrations) [185].

Besides the effects of SeNPs per se, these particles can be used for the delivery of specific drugs. The size of these materials allows for efficient uptake by target cells and drug accumulation at target sites [186]; moreover, they can be decorated to enhance anticancer efficacy through increased uptake [187,188].

In this regard, the chemotherapeutic agent doxorubicin has been loaded into diselenide polymeric micelles to be released in cancer cells [189], and hydrogel and metal-organic frameworks (MOFs) have also been developed. In this frame, hydrogels responsive to radiation applied as radiotherapy that produces ROS (gamma radiation), cleaving diselenide bonds, have been projected to load anticancer drugs. Upon the exposure of both micelles and hydrogels to irradiation, the nanoparticles disassemble (due to the cleavage of the diselenide bond), and the chemotherapeutic drug is released.

Concerning monoselenide, also in this case, micelles formed by selenium-containing polymers can be loaded with drugs (e.g., doxorubicin) and are sensitive to oxidation stimuli [190], such as treatment with hydrogen peroxide or exposure to near-infrared light, and can revert and reassemble under reductive stimuli.

Selenium-containing nanomaterials can be used as drug carriers, but they show an anticancer effect due to the presence of selenium *per se*.

Selenium shows antioxidant properties at low levels, while it becomes a pro-oxidant at high doses, increasing ROS production. Although cancer cells show high levels of ROS and reducing agents, they are sensitive to additional ROS; this reflects an ability of selenium to impair cancer cell survival. Moreover, the diselenide-containing polymer has been proposed as a drug carrier able to include hydrophobic anticancer compounds, while monoselenide-containing polymers possess anticancer activity independent of the ability to encapsulate drugs [191]. Recently, great interest was given to developing selenium-containing nanomaterials to be applied both for the delivery of anticancer drugs and for showing anticancer activity *per se*. In a work by Zhang and collaborators, the authors describe a very innovative approach for the delivery of the chemotherapeutic agent paclitaxel, taking advantage of spherical nanoparticles containing diselenide-containing small molecules, and they show the effectiveness of these chimeric particles in inducing apoptosis selectively in cancer cells (e.g., HeLa and MCF-7) through the generation of ROS [135]. Coherently, diselenide-containing nanocapsules have been reported to exert anticancer effects due to the activity of both selenium and encapsulated drugs [192].

### 5.3. Therapeutic Potential of SeNPs Per Se

Notably, other interesting publications report that coordination complexes (e.g., coordinating platinum) containing selenium have anticancer effects being highly toxic against cancer cells (both in vitro and in vivo) in a selective way due to the induction of ROS and above all to the presence of selenium [136,137]. The selenium-containing selenomethionine (**2**)-based self-assemblies exert anticancer abilities [193] and can be combined with drugs to deliver specific compounds in cancer cells. In addition to platinum, selenium can interact with other metals (e.g., gold, zinc, copper, nickel) to constitute nanoparticles with anticancer activity through the induction of ROS in cancer cells, stimulating their apoptosis [194]. Selenium-containing molecules can also be combined with porphyrins [195,196], can be activated by light (photodynamic therapy) and can activate cell apoptosis. Moreover, selenium-containing polymers activate the immune response by NK cells, thus representing a synergic strategy with low side effects to apply for tumour management in combination with conventional therapies [138]. In order to increase SeNPs’ solubility, stability, cellular uptake (also increased by the nanoparticles’ decoration with conventional chemotherapeutic agents such as 5-fluorouracil [187]) and efficacy against cancer cells in vivo and in vitro, chemical strategies have been developed, such as the addition of polysaccharides (e.g., spirulina [188,197]), proteins (e.g., transferrin [198]), folic acid [199] or hydrogels [200] coating them. Despite the described role of SeNPs to facilitate drug delivery, they can be considered as shuttles for other small molecules such as siRNAs targeting specific cancer-related mRNAs [201] or viral RNAs [202].

### 5.4. Therapeutic Potential of SeNPs in Non-Cancer Diseases

SeNPs have been applied not only to cancer but also to other diseases of different origins. One of the recent examples of the application of SeNPs is the treatment of *Vibrio cholerae* infection. Notably, selenium particles increase the immune responses in vivo in a mouse cholera model due to their cytotoxic activity and to a significant increase in *V. cholerae*-specific IgG and IgA responses in serum and saliva. Moreover, the levels of both IL-4 and IL-5 are significantly increased, indicating that SeNPs induce immune cell effectors [139].

In this regard, apoptosis is a common pathway for destroying intracellular bacteria [203] that prevents pathogens from exiting the host cells for further infection spread. However, some pathogens possess highly stable cell walls and the ability to inhibit host cell apoptosis as a critical way for their immune escape. Notably, a recent work on Mtb infection reported the ability of SeNPs to induce infected macrophage apoptosis, which is useful for intracellular clearance and antimicrobial immunity [173].

Moreover, SeNPs can be used to counteract multidrug-resistant bacterial infections [204] and viral infections (e.g., type-1 dengue virus [205]), both alone and in combination with lysozyme [206].

Gram-negative, Gram-positive, multiresistant bacteria, *C. albicans*, different protozoa and parasites (e.g., *Leishmania*, *Toxoplasma gondii* and *Schistosoma mansoni*), and several viral infections (e.g., influenza, hepatitis, HIV, H1N1) have been reported as sensitive targets of SeNPs [207].

This approach is also helpful in tumour cells where SeNPs activate the early phase of autophagy while the late one is blocked [208].

Moreover, SeNPs display a protective role against the cytotoxic effects of several chemotherapeutic agents such as cisplatin [209]. In this regard, the co-treatment with both cisplatin and SeNPs increases serum testosterone, sperm quality, and spermatogenesis and limits nephrotoxicity [210].

### 5.5. Advantages of SeNPs in Cancer Treatment

The high bioavailability (depending overall on particle size [211]) together with the low toxicity (lower than selenomethionine [212]) make the nanoform of selenium more attractive with respect to the inorganic and organic forms [212,213,214]. Moreover, SeNPs can resist adverse conditions such as digestion and enzymatic cleavage and use the selenium in a zero oxidation state, which is low toxic, bioavailable [211,212] and stable when encapsulated inside particles such as chitosan (CS) [215]. In this regard, the incorporation of selenium into CS nanoparticles improves the availability of this element, facilitates the expression of selenoproteins and can be translated into therapy as a chemopreventive agent and chemotherapeutic drug delivery approach [184,187,216,217].

Many literature reports demonstrate that selenium can both prevent/reduce cancer incidence [218,219] and can act in synergy with chemotherapeutics [220,221].

The dual role of SeNPs as anticancer agents and drug carriers has been extensively investigated [188,222,223,224,225,226] highlighting their ability to impair cancer cell growth [216,227]. Moreover, SeNPs are able to enter cancer cells and show high ability in chemoprevention, inducing glutathione S-transferase (GST) more than SeMet and selenite [212,228].

Notably, a large spectrum of experimental evidence obtained in different tumour cells highlights the promising role of SeNPs in inducing mitochondria-mediated apoptosis [185] in melanoma cells, cell cycle progression inhibition in pancreatic cancer cells, antiproliferative effects in human cervical carcinoma and breast cancer cells, and anticancer activity in hepatocellular carcinoma cells [183,229,230]. Focusing on cell proliferation and other typical features of tumour cells such as viability, cell cycle arrest, migration and invasion, it has been reported in an in vitro model of hepatocellular carcinoma that the effects of SeNPs and selenocysteine were similar, while Se + IV was more toxic and cells treated with Se + IV, selenomethionine and seleno-metylselenocysteine did not show significant difference with respect to not-treated ones.

These direct effects of SeNPs on tumour cells parallel with their potential as chemotherapeutic delivery carriers [183,187,231,232,233,234,235] since they passively accumulate in cancer cells, and their decoration on the surface enhances their specific uptake [188,199]. The SeNPs activate ROS and, in turn, regulate the apoptotic signal mediated by p53 and MAPK phosphorylation, also preventing DNA repair in cancer cells [168]. Among the therapeutic cargo described for SeNPs, 5-fluorouracil and cisplatin are the best-known drugs [187].

In addition to cell proliferation/invasion/migration, SeNPs are also effective against cancer cells by stimulating the immune response [236] due to the increase in chemotactic activity and respiratory burst response of neutrophils [237]; this effect was not evident after the treatment with inorganic selenium.

Despite the growing interest of scientists in SeNPs and the high number of reports on their wide range of positive effects, there are some concerns about their toxicity and objections to their use in clinical practice. Based on the described formulations of selenium to be applied in therapy, it seems evident that not only the concentration of this element but also the chemical form is crucial for its effectiveness against several diseases, including cancer [187]. Specifically, with respect to the SeNPs, they clearly show a notable advantage due to their low grade of toxicity, higher efficiency and biosafety [212,238,239] compared with selenite (LD_50_ around 18-fold higher), chemopreventive potential [187] and selectivity against cancer cells.

## 6. Safety of Selenium-Containing Compounds

Despite the encouraging results about the role of Se-containing compounds as chemopreventive and anticancer agents, the clinical application is sometimes limited owing to their possible side effects [82]. Mainly, inorganic compounds are known to be metabolised into redox-active products, such as hydrogen selenide, which arises from selenite **3**, whose accumulation into cells can lead to genotoxic effects (single strand DNA breaks), likely as a consequence of the oxidative stress induction [52]. For this reason, inorganic Se-containing compounds are considered more toxic than organic ones [52]. Furthermore, sodium selenite **3a** resulted in being well tolerated in short-term toxicity studies [52].

In the last few years, significant attention has been devoted to Se-containing organic molecules to retain the pharmacological properties of inorganic compounds while lowering their toxicity risk. Notably, selenoaminoacids, such as selenomethionine **2** (L-selenomethionine) and methylselenocysteine **1**, exhibited a very low toxicity and lack genotoxicity [52,240]; moreover, methylseleninic acid **6** was found to be nongenotoxic with respect to selenite **3** and nontoxic to normal tissues [40].

Diphenyl diselenide **8** showed a toxicity risk in vivo, depending on the route of administration and dosage, but its derivatives, among which includes DSBA **8a**, could overcome this limit [51]. Accordingly, selol resulted to be nontoxic and nonmutagenic only when administered parenterally; by contrast, it was toxic after oral administration, likely due to the formation of harmful metabolites during digestion [52].

Overall, the mechanisms involved in selenium toxicity are not entirely understood yet; however, the oxidation of thiols (e.g., glutathione and cysteine) by both inorganic and organic selenium-containing compounds seems to play a central role in their toxicity as well as in their biological activities [241]. Considering that even compounds with low toxicity in vivo (e.g., ebselen **4** and diphenyl diselenide **8**) can determine toxic effects at high doses, depending on the species, exposure time, and route of administration, it is of utmost importance to support the pharmacological findings with toxicological studies in order to allow for future clinical application of these bioactive compounds.

## 7. Conclusions

In this review, we illustrate the most promising Se-containing compounds and SeNPs that have emerged as promising cancer treatments in recent years.

In comparison to conventional chemotherapeutic agents, organoselenium compounds such as **10–14** have excellent cytotoxic properties with low side effects [99,102,105,106,109]. Recent studies have also pointed to selenium-based nanomedicines, suggesting the design of selenium nanoparticles as an efficient strategy to improve drug delivery and thus cancer treatment [190].

Over the last decade, significant progress has been made in understanding the complex biology and chemistry of Se. It was long believed that Se and Se-containing compounds maintain the redox status of cells and prevent oxidative damage caused by ROS. Recent studies indicate, however, that Se plays a dual role in oxidative stress, exhibiting a pro-oxidant and an antioxidant function depending on the type of incorporation of Se in the relative compound. The uncertainty about the anticancer and immunostimulatory properties of Se agents is often also reflected in their unclear mechanism [2]; thus, more research is needed.

As discussed above, some compounds are rather promiscuous binders and, as such, can only be considered as hit compounds needing further optimization. An excellent example of a rather advanced Se-containing compound is compound BSP16 (**15**, LF250), a selenium-containing STING agonist, with excellent anticancer properties in vitro and in vivo better than its sulphur containing analogue. Furthermore, it possesses an excellent pharmacokinetic profile as an oral drug leading to tumour regression after 21 days in a colon carcinoma tumour model [118].

However, most other Se-containing agents in our collection are not (yet) very potent when it comes to in vivo efficacy.

Besides a direct anti-cancer action of Se-containing agents, it might also be interesting to apply a hybrid approach as shown by Ali et al. [102,242]. The phenylselenoethers exhibited P-glycoprotein (ABCB1) MDR efflux pump inhibitory activity and possessed interesting cytotoxic and antiproliferative MDR mouse T-Lymphoma cells reaching, with compound **11**, the submicromolar range. Notably, compound **11** was also more active when co-administered with the well-known chemotherapeutic agent doxorubicin, underlining the fact that a combination therapy approach is worthwhile to investigate.

Due to the small number of researchers and companies working on these promising agents, the optimization process is relatively slow [4]. An interdisciplinary effort is urgently required to dissect the biological effects of the different Se incorporations in small organic molecules in order to improve the situation, thereby opening the door to the development of more highly potent and target-specific Se-containing agents. These molecules seem to possess essential advantages: high specificity and the high effectiveness, above all, in relation to their formulation that can include the Se element in low-toxic molecules or can insert Se inside NPs. In our view, researchers should try more often to replace oxygen or sulphur with Se in their design strategies for novel anti-cancer small molecules. In addition, the approach to create SeNPs has promising first data in cancer, but intensive research is necessary from a toxicity point of view prior to thinking of an application in therapy.

To sum up, Se-based therapy approaches in cancer might have a bright future, but a lot of research efforts are still necessary prior to seeing a clinical candidate or even an approved drug.

## Figures and Tables

**Table 1:** The main health problems connected with selenium deficiency or excess [19,20,21].

Condition	Health Problem	Concentration in Plasma
deficiency	Keshan and Kashin–Beck disease immune disorders decrease fertility (in men) thyroid autoimmune diseases cognitive decline/dementia (e.g., AD, PD) *type 2 diabetes* cancer (e.g., *prostate cancer*) weaker defence against infectious diseases (e.g., HIV, hepatitis C, influenza A, Ebola or SARS-CoV-2) *increased mortality*	<85 µg/L
optimal level	Good health	90–120 µg/L
excess	selenosis alopecia dermatitis *increased mortality* *type 2 diabetes* *prostate cancer risk*	>125 µg/L *

* potentially toxic dose taken as a dose above the optimal dose; *in italics*—health problems related to both the excess and the deficit of Se.

## Data Availability

No additional data has been created.
